# Interaction between ZnO Nanoparticles and Albumin and Its Effect on Cytotoxicity, Cellular Uptake, Intestinal Transport, Toxicokinetics, and Acute Oral Toxicity

**DOI:** 10.3390/nano11112922

**Published:** 2021-10-31

**Authors:** Eun-Been Jung, Jin Yu, Soo-Jin Choi

**Affiliations:** Division of Applied Food System, Major of Food Science & Technology, Seoul Women’s University, Seoul 01797, Korea; ebe2@swu.ac.kr (E.-B.J.); ky5031@swu.ac.kr (J.Y.)

**Keywords:** zinc oxide, food protein, interactions, cytotoxicity, cellular uptake, intestinal transport, toxicokinetics, acute oral toxicity

## Abstract

Zinc oxide (ZnO) nanoparticles (NPs) are used as zinc supplements due to the nutritional value of Zn. The toxicity of ZnO NPs in the food industry is required to be elucidated because they have large surface area and high reactivity compared with bulk-sized materials and have potentials to interact with food matrices, which may lead to different biological responses. In this study, interactions between ZnO NPs and food proteins (albumin, casein, and zein) were evaluated by measuring changes in physicochemical property, fluorescence quenching ratios, and structural protein stability compared with ZnO interaction with glucose, the most interacted saccharide in our previous report. The interaction effects on cytotoxicity, cellular uptake, intestinal transport, toxicokinetics, and acute oral toxicity were also investigated. The results demonstrate that interaction between ZnO and albumin reduced hydrodynamic diameters, but increased cytotoxicity, cellular uptake, and intestinal transport in a similar manner to ZnO interaction with glucose, without affecting primary structural protein stability and toxicokinetic behaviors. Hematological, serum biochemical, and histopathological analysis reveal no toxicological findings after orally administered ZnO NPs interacted with albumin or glucose in rats for 14 consecutive days, suggesting their low oral toxicity. In conclusion, the interactions between ZnO NPs and food proteins modulate in vitro biological responses, but do not affect in vivo acute oral toxicity. Further study is required to ascertain the interaction effects on chronic oral toxicity.

## 1. Introduction

Zinc oxide (ZnO) has multifunctionality due to its semi-conductor, optical, biological, and antibacterial properties [1]. ZnO is also applied to various commercial foods as a Zn supplement or agricultural fertilizer due to nutrient value and diverse biological functions of Zn [2,3,4,5]. ZnO is an essential mineral for the body and a generally recognized as safe (GRAS) material. However, high level of Zn causes nausea, vomiting, stomach pain, diarrhea, flu-like symptoms, and other nutritional deficiency [6,7,8]. Nanotechnology development has led to the production of ZnO nanoparticles (NPs) in the size range of 1–100 nm, which can change physicochemical properties and biological responses of ZnO compared with those of bulk-sized materials. Moreover, food additive ZnO NPs are added directly to foods consisting of proteins, carbohydrates, minerals, and other components. Hence, interactions between ZnO NPs and food matrices can easily occur, which may lead to different physicochemical, biological, and toxicological effects compared with those of pristine ZnO NPs [9,10,11].

Remarkably reduced hydrodynamic diameters of silicon dioxide (SiO_2_) and titanium dioxide (TiO_2_) NPs and their high oral absorption in rats were found when SiO_2_ and TiO_2_ NPs were dispersed in glucose and serum albumin, respectively, compared with those of NPs in distilled water (DW) [12,13]. These results suggest that the interactions between NPs and glucose or serum albumin could reduce the hydrodynamic diameters of NPs by increasing dispersion stability, consequently contributing towards enhancing their in vivo oral absorption. The role of NP–protein interaction, protein corona, in the immune system was also emphasized [14,15,16,17]. The lipid and protein corona of food additive TiO_2_ NP in an in vitro simulated gastrointestinal digestion fluid was demonstrated, which reduced oxidative stress and cytotoxicity in cell lines [18]. Interactions between ZnO NPs and saccharide matrices, such as fructose, glucose, sugar mixture, and acacia honey containing high levels of saccharides, were quantitatively determined in our previous reports [9]. Among them, glucose was found to significantly enhance cellular uptake, intestinal transport, and in vivo oral absorption of ZnO NPs [9]. On the other hand, a contradictory result was reported, showing that a high protein-containing complex food (skim milk) or its main components, casein and lactose, had no effect on cytotoxicity, cellular uptake, or intestinal transport of ZnO NPs [10]. Therefore, the effects of interactions between ZnO NPs and food matrices on in vitro and in vivo biological responses are required to be investigated more extensively to predict and understand potential toxicity of food-additive NPs.

We hypothesized that ZnO NPs can interact with food proteins, which may modulate in vitro and in vivo biological responses. In this study, interactions between ZnO NPs and food proteins, such as albumin from chicken egg white, casein from bovine milk, and zein from corn, were evaluated in terms of physicochemical properties, protein fluorescence quenching, structural protein stability, cytotoxicity, cellular uptake, intestinal transport, and in vivo oral absorption. Comparative study with glucose, the most interacted saccharide with ZnO NPs in our previous report [9], was also carried out. Finally, in vivo oral toxicity study was performed after oral administration of ZnO NPs interacted with proteins or glucose in rats for 14 consecutive days to answer the question whether the interactions affect acute oral toxicity of ZnO NPs.

## 2. Materials and Methods

### 2.1. Materials and NP Preparation

ZnO NPs (product number 544906, particle size < 100 nm, specific surface area 10–25 m^2^/g, purity > 95%), albumin from chicken egg white, casein salt from bovine milk, zein, D– (+)–glucose, ethylenediaminetetraacetic acid disodium salt dihydrate (EDTA), pepsin from porcine gastric mucosa, and β-mercaptoethanol were purchased from Sigma-Aldrich Co. Ltd. (St. Louis, MO, USA). Sodium dodecyl sulfate (SDS) and Tris were provided by Duchefa Biochemie (Haarlem, The Netherlands). Acrylamide-bis solution (29:1) and 0.1% bromophenol blue were supplied from Bio-Rad Laboratories Inc. (Hercules, CA, USA), and Daejung (Siheung, Gyeonggi-do, Korea), respectively. Ammonium persulfate, ethyl alcohol, nitric acid (HNO_3_), hydrogen peroxide (H_2_O_2_), hydrogen chloride (HCl), and glycerol were supplied by Samchun Pure Chemical Co., Ltd. (Pyeongtaek, Gyeonggi-do, Korea). Minimum essential medium (MEM), Dulbecco’s modified Eagle’s medium (DMEM), Roswell Park Memorial Institute (RPMI) 1640, inactivated fetal bovine serum (FBS), penicillin, streptomycin, Dulbecco’s phosphate-buffered saline (DPBS), and phosphate-buffered saline (PBS) were purchased from Welgene Inc. (Gyeongsan, Gyeongsangbuk-do, Korea). Water-soluble tetrazolium salt (WST-1) and 2′,7′-dichlorofluorescein diacetate (H_2_DCFDA) were provided by Roche (Mannheim, Germany) and Molecular Probes Inc. (Eugene, OR, USA), respectively. A CytoTox 96 Nonradioactive Cytotoxicity Assay kit was obtained from Promega (Madison, WI, USA). Matrigel from Corning Inc. (Corning, NY, USA) and Transwell polycarbonate inserts from SPL Life Science Co., Ltd. (Pocheon, Gyeonggi-do, Korea) were used.

Stock suspension of ZnO NPs (5 mg/mL) was prepared in DW, MEM, casein (1 mg/mL), albumin (1 mg/mL), zein (1 mg/mL), or glucose (1 mg/mL) solutions by stirring for 30 min, followed by bath sonication (160 Watts, Bransonic 5800, Branson Ultrasonics, Danbury, CT, USA) for 5 min prior to all experiments. Zein, a water insoluble protein, was dissolved in 94.5% ethanol at 10 mg/mL and diluted to 1 mg/mL with DW or MEM before experiments.

### 2.2. Characterization

The constituent particle size and shape of ZnO NPs were determined by field emission–scanning electron microscopy (FE–SEM; JSM-7100F, JEOL, Tokyo, Japan). Particle size distribution was measured by randomly selecting more than 100 particles from the SEM images. Hydrodynamic diameters and zeta potentials of ZnO NPs prepared in DW, MEM, and each food matrix solution were measured via dynamic light scattering (DLS) and electrophoretic light scattering, respectively, using a Zetasizer Nano System (Zetasizer Nano ZS, Malvern Instruments, Worcestershire, UK) after dilution with DW and MEM, respectively.

### 2.3. Dissolution Properties of ZnO NPs

The effect of the interactions between ZnO NPs and food proteins on the solubility of ZnO NPs was investigated by dispersing ZnO NPs (5 mg/mL) interacted with food proteins in DW, MEM, or artificial lysosomal fluid (ALF, Table S1) with shaking at 37 °C [19]. After 0.5, 1, 6, and 24 h, the supernatants were collected by ultracentrifugation (16,000× *g*) for 15 min. Solubility of ZnO NPs in in vitro simulated digestion models (Table S2) was also evaluated by dispersing ZnO NPs (5 mg/mL) interacted with food proteins in simulated saliva, gastric fluid, and intestinal fluids for 5 min, 2 h, and 2 h, respectively, on a head-over-head rotator at 37 °C [20]. For solubility in three consecutive digestion fluids, ZnO NPs (5 mg/mL) were dispersed in 6 mL of simulated saliva for 5 min at 37 °C, followed by digestion in 12 mL of gastric fluid for 2 h at 37 °C. Finally, the digestion was carried out for a further 2 h at 37 °C after addition of 12 mL of duodenal fluid and 6 mL of bile fluid to the suspension. After the samples were centrifuged at 16,000× *g* for 15 min, quantitative analysis of the dissolved Zn from ZnO in the supernatants was carried out using inductively coupled plasma–atomic emission spectroscopy (ICP-AES; JY2000 Ultrace, HORIBA Jobin Yvon, Longjumeau, France), as described in “2.4. ICP–AES Analysis”. ALF and digestion fluids were prepared on the day of the experiment at 37 °C.

### 2.4. ICP–AES Analysis

All samples were digested with 10 mL of ultrapure HNO_3_ at ~160 °C, and 1 mL of H_2_O_2_ solution was added and heated until the samples were colorless and clear. After digestion, the samples were diluted with distilled and deionized water (DDW), and total Zn concentrations were determined by ICP–AES (JY2000 Ultrace, HORIBA Jobin Yvon, Longjumeau, France).

### 2.5. Fluorescence Quenching of Food Proteins by ZnO NPs

Interactions between ZnO NPs and food proteins were investigated by measuring fluorescence quenching ratios. ZnO NPs (0.5 mg/mL) were suspended in casein (1 mg/mL), albumin (1 mg/mL), or zein (1 mg/mL) and incubated with gentle shaking at 25 °C. After incubation for 0.02, 0.5, 1, 6, and 24 h, the suspensions were transferred to quartz cuvettes and protein fluorescence quenching was analyzed using a luminescence spectrometer (SpectraMax M3, Molecular Devices, Sunnyvale, CA, USA). The excitation wavelength was set at 280 nm and fluorescence emission was scanned at wavelengths from 300 to 420 nm. Fluorescence quenching ratios were calculated according to the equation: (I_0_ − I)/I_0_, where I_0_ and I are the fluorescence intensity of proteins in the absence and presence of ZnO NPs, respectively.

### 2.6. SDS–Polyacrylamide Gel Electrophoresis (PAGE) Analysis

Changes in the primary structure of food proteins by interaction with ZnO NPs were analyzed by SDS–PAGE. Casein, albumin, or zein (1 mg/mL) were incubated with ZnO NPs (0.5 mg/mL) by gentle shaking at 25 °C for 0.02, 0.5, 1, 6, and 24 h.

Effect of the interactions on the digestion of food proteins was evaluated by pepsin treatment. Pepsin at final concentration of 1990 units/mL was added to ZnO NPs–protein suspensions and their pH were adjusted to 1.5 with 1 N HCl. After incubation for 1 h at 37 °C with gentle shaking, the samples were concentrated using a nitrogen evaporator (MG-3100, Eyela, Tokyo, Japan). All protein samples were re-suspended in DW (1 mL) and concentration of proteins were determined using Bradford reagent (Bio-Rad, Hercules, CA, USA).

Protein samples were diluted in sample buffer, 50 mM Tris-HCl (pH 6.8) containing 4% SDS, 0.1% bromophenol blue, 10% glycerol, and 0.5% β–mercaptoethanol, and heated at 95 °C for 5 min. After cooling to room temperature, samples were loaded onto 14% SDS-polyacrylamide gels and electrophoresis was performed with a power supply (MP–3AP, Major science, Saratoga, CA, USA) under a voltage of 150 V for 2 h.

### 2.7. Cytotoxicity

#### 2.7.1. Cell Culture

Human intestinal epithelial Caco-2 cells and human Burkitt’s lymphoma Raji B cells were purchased from the Korean Cell Line Bank (Seoul, Korea). The cells were cultured in MEM and RPMI, respectively, supplemented with 10% FBS, 100 units/mL of penicillin, and 100 µg/mL of streptomycin in a 5% CO_2_ incubator at 37 °C.

#### 2.7.2. Cell Proliferation

The interaction effect on cell proliferation was measured with WST–1 (Roche). Cells (1 × 10^4^ cells/100 µL) were treated with ZnO NPs suspended in DW, MEM, or each food matrix solution. All food matrices (1 mg/mL) for cell experiments were prepared in MEM by stirring for 30 min. After incubation for 24 h, 10 µL of WST-1 solution was added to each well and further reacted for 4 h. The absorbance was measured at 440 nm versus 650 nm using a microplate reader (SpectraMax M3, Molecular Devices, Sunnyvale, CA, USA).

#### 2.7.3. Lactate Dehydrogenase Leakage (LDH) Assay

The released levels of LDH were measured with the CytoTox 96 Non-Radioactive Cytotoxicity assay (Promega, Madison, WI, USA). Cells (1 × 10^4^ cells/1 mL) were treated with ZnO NPs suspended in DW, MEM, or each food matrix solution. After 24 h, the medium was collected and centrifuged, and 50 µL of the supernatants were treated with a substrate solution (50 µL) for 30 min in the dark at room temperature. Finally, 50 µL of a stop solution was added and the absorbance was measured at 490 nm with a microplate reader (SpectraMax M3, Molecular Devices).

#### 2.7.4. Reactive Oxygen Species (ROS) Generation

The intracellular levels of ROS were evaluated using a peroxide-sensitive fluorescent probe, H_2_DCFDA. Cells (1 × 10^4^ cells/100 µL) were incubated with ZnO NPs dispersed in DW, MEM, or each food matrix solution. After 24 h, 20 µM H_2_DCFDA was added to each well and further incubated for 30 min in the dark at 37 °C. Finally, the wells were washed with DPBS and 2′,7′-dichlorofluorescein (DCF) fluorescence was immediately measured by a fluorescence microplate reader (SpectraMax M3, Molecular Devices, Sunnyvale, CA, USA). The excitation and emission wavelengths were set at 485 nm and 535 nm, respectively.

### 2.8. Cellular Uptake

Cells (1 × 10^6^ cells/2 mL) were incubated with ZnO NPs (50 µg/mL) prepared in DW, MEM, or each food matrix solution. After 6 h, the cells were washed three-times with DPBS and treated with 5 mM EDTA for 40 s to detach adsorbed NPs on the membrane surface. After washing with DPBS three-times, the cells were harvested with a scraper, centrifuged, and re-suspended in DDW. Total intracellular Zn levels were analyzed as described in “2.4. ICP–AES Analysis”. The cells in the absence of NPs were used as a control.

### 2.9. Intestinal Transport

A follicle-associated epithelial (FAE) model, mimicking microfold (M) cells, was prepared as previously described [10,21]. Transwell polycarbonate inserts were coated with Matrigel matrix prepared in serum-free DMEM for 2 h and washed with serum-free DMEM. Caco-2 cells (1 × 10^6^ cells/well) were cultured on apical sides and grown for 14 days. Lymphoma Raji B cells (1 × 10^6^ cells/well) were added to the basolateral sides and the co-cultures were maintained for 5 days until trans epithelial electrical resistance (TEER) values reached 150–200 Ω cm^2^. The apical medium of co-culture monolayers was replaced with ZnO NPs (50 µg/mL) prepared in DW, MEM, or each food matrix solution and incubated for 6 h.

Caco-2 monoculture was also used to evaluate the transport of ZnO NPs by the intestinal epithelial tight junction barriers. Caco-2 cells (4.5 × 10^5^ cells/well) were cultured on apical sides for 21 days (TEER ≥ 300 Ω cm^2^). The apical medium of the monolayers was replaced with ZnO NPs (50 µg/mL) prepared in DW, MEM, or each food matrix solution and incubated for 6 h. Finally, the basolateral solutions were collected and analyzed as described in “2.4. ICP–AES Analysis”. The FAE and Caco-2 monolayer models in the absence of ZnO NPs were used as controls.

### 2.10. Animals

Six-week-old female Sprague-Dawley SD rats weighing 170–180 g were purchased form Koateck (Pyeongtaek, Gyeonggi-do, Korea). Rats were housed in laboratory animal cages placed in a ventilated rack maintained at 20 ± 2 °C and 60 ± 10% relative humidity with a 12 h light/dark cycle. Water and commercial laboratory complete food for rats were given ad libitum. Animals were environmentally acclimated for 7 days before administration. All animal experiments were conducted in accordance with guidelines for the Institutional Animal Care and Use Committee (IACUC) of the Seoul Women’s University. Protocols used in this study were approved by the Seoul Women’s University IACUC (SWU IACUC-2019A-1).

### 2.11. Toxicokinetic Study

Six female rats per group (~200 g) were administered a single dose (100 mg/kg) of ZnO NPs dispersed in DW, 5% albumin, or 5% glucose via oral gavage. Blood samples from the tail veins of the rats were collected at 0, 0.25, 0.5, 1, 2, 4, 6, 10, and 24 h after administration, and centrifuged at 3000× *g* at 4 °C for 15 min to obtain the plasma. The total plasma Zn concentrations were analyzed as described in “2.4. ICP–AES Analysis”. The following toxicokinetic parameters were assessed using pharmacokinetic modeling program (version 1.03.35, APL, Eden Prairie, MN, USA): maximum concentration (C_max_), time to maximum concentration (T_max_), area under the plasma concentration–time curve (AUC), half-time (T_1/2_), mean residence time (MRT), and oral clearance (Cl/F).

### 2.12. 14-Day Repeated Oral Toxicity Study

Five female rats per group were administered ZnO NPs (100 mg/mL) dispersed in DW, 5% albumin, or 5% glucose via oral gavage for 14 consecutive days. An equivalent volume of DW was also administered in rats as a control. Changes in body weight, food intake, and water consumption were recorded daily, and abnormal symptoms and behaviors were observed after oral administration. At the end of the experiment, all animals were euthanized by CO_2_ and organs including brain, heart, kidney, large intestine, liver, lung, ovary, small intestine, spleen, and stomach were collected. Organo-somatic indices were calculated by the following formula: weight of the organ (g)/total body weight (g) × 100. Blood samples were collected from the posterior vena cava for hematological and serum biochemical analysis as previously described [22]. Hematological, aggregation time, and biochemical analysis were performed by using automatic hemato-analyzer (ADVIA 2120i, Siemens, Tarrytown, NY, USA), coagulometer (ALC 7000, Werfen Medical, IL, USA), and biochemical analyzer (TBA-120FR, Toshiba, Otawara, Japan), respectively. Organs including kidneys, liver, lungs, and spleen were fixed with 10% neutral buffered formalin and stained with hematoxylin and eosin for histopathological examination.

### 2.13. Statistical Analysis

All experimental data were presented as means ± standard deviations. One-way analysis of variance with Tukey’s test in SAS version 9.4 (SAS Institute Inc., Cary, NC, USA) was performed to determine the significances of intergroup differences. Statistical significance was accepted for *p* values of < 0.05.

## 3. Results and Discussion

### 3.1. Characterization of ZnO NPs in Food Proteins

**Figure 1:**
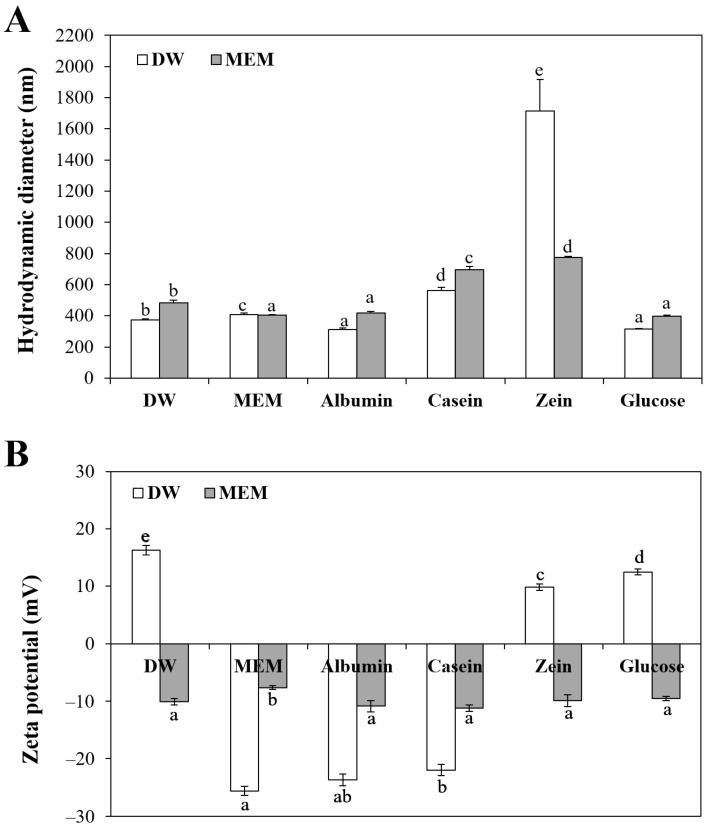
(**A**) Hydrodynamic diameters and (**B**) zeta potentials of ZnO NPs in food proteins or glucose, as measured by dilution in distilled water (DW) or cell culture minimum essential medium (MEM). Different lowercase letters (a–e) indicate significant differences among different matrices (*p* < 0.05).

The constituent particle sizes of ZnO NPs were determined to be ~78 ± 26 nm with irregular round or oval shapes by SEM analysis (Figure S1). The hydrodynamic diameters and zeta potentials of ZnO NPs in the presence of food proteins, glucose, or MEM are presented in Figure 1. Glucose was included for comparative study because our previous report demonstrated that ZnO NPs in glucose had higher cellular uptake, intestinal transport, and in vivo oral absorption than those of ZnO NPs in other saccharide matrices [8]. Cell culture medium, such as MEM, was reported to play a role in NP dispersion and ZnO NPs in MEM also exhibited efficient cellular uptake and intestinal transport [9,23,24]. The hydrodynamic diameters of ZnO NPs were measured after interaction with food proteins, glucose, or MEM, followed by dilution in DW or MEM. Figure 1A shows that the hydrodynamic diameters of ZnO NPs increased compared with constituent particle sizes (Figure S1), indicating their aggregate fate under aqueous conditions. The role of MEM as an NP dispersant was not remarkable, except ZnO NPs in zein, a highly insoluble protein in water [25,26]. Among food proteins, ZnO NPs in albumin showed the smallest hydrodynamic diameters, whereas ZnO NPs were found to be highly aggregated by interaction with casein or zein. It is worth noting that ZnO NPs interacted with glucose had the same hydrodynamic diameters to those of ZnO NPs in albumin when DLS was measured after dispersion in both DW and MEM. These results suggest that the hydrodynamic diameters of ZnO NPs could be highly affected by interaction with food matrices.

Zeta potential values of ZnO NPs differed from matrix types when they were measured in DW (Figure 1B). The zeta potentials of ZnO NPs in DW were 16.3 ± 0.8 mV but became negative when ZnO NPs were interacted with albumin or casein. Indeed, isoelectric points (IEPs) of albumin and casein are 4.9 and 4.6, respectively. Hence, negative charges of albumin and casein at neutral pH seem to change zeta potential values of ZnO NPs to negative charges by the interactions. On the other hand, zein with IEP at 6.2 had minor effect on zeta potential of ZnO NPs, changing slightly to less positive charge (9.8 ± 0.6 mV). It is worth noting that positive charge of ZnO NPs in DW became negative regardless of food matrix types when their zeta potentials were measured in MEM. This can be explained by IEP (pH 4.5–5.0) of bovine serum albumin present in MEM [23,27,28]. Therefore, the interaction effect on the zeta potentials of ZnO NPs under cell culture condition seems to be minor.

### 3.2. Solubility of ZnO NPs in Food Proteins

**Figure 2:**
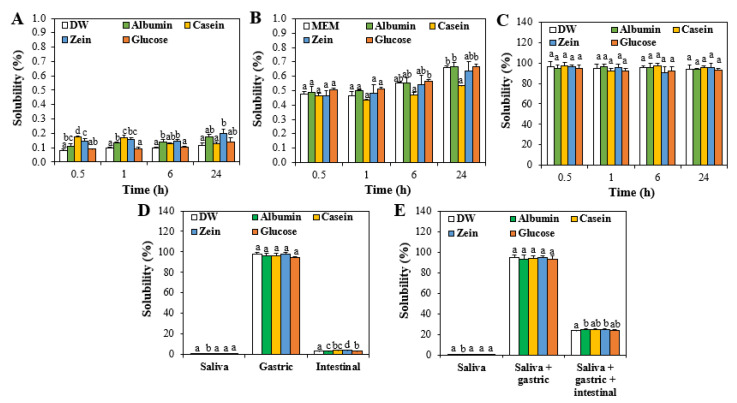
In vitro solubilities of ZnO NPs (5 mg/mL) interacted with food proteins or glucose in (**A**) distilled water (DW), (**B**) cell culture minimum essential medium (MEM), (**C**) artificial lysosomal fluid (ALF), (**D**) simulated digestion fluids, and (**E**) in vitro three consecutive digestion fluids at 37 °C. Different lowercase letters (a–d) indicate significant differences among different matrices (*p* < 0.05).

The solubilities of ZnO NPs interacted with food proteins or glucose increased in order of those in DW < MEM < ALF (Figure 2A–C). High solubility of ZnO NPs in ALF (pH 4.5) is likely to be associated with their rapid dissolution property under acidic condition [29,30,31,32]. On the other hand, the solubilities of ZnO NPs in in vitro digestion fluids such as saliva, gastric, and intestinal fluids were highly different (Figure 2D). The solubilities of ZnO NPs in saliva and intestinal fluid were low, but increased dramatically to 98% in the gastric fluid. These results can also be explained by low and high dissolution properties of ZnO under alkaline and acidic conditions, respectively [30,32,33]. Slightly but significantly increased solubilities were found by the interactions between ZnO NPs and food proteins in the intestinal fluid, although the solubilities are low (3.4% in DW versus 3.8–4.5% in matrices).

When the solubilities of ZnO NPs was evaluated in three consecutive digestion fluids, their extremely low dissolution (~0.1%) in saliva, high dissolution in saliva followed by gastric fluid up to ~95%, and ~25% dissolution in saliva followed by gastric and intestinal fluids were found (Figure 2E). The interaction effect on the solubility was not remarkable. High solubility of ZnO NPs in the gastric fluid is surely attributed to their high dissolution property in acids, as observed in their high solubility in ALF (Figure 2C). Meanwhile, decreased solubility of ZnO NPs in three consecutive fluids compared with that in saliva followed by gastric fluid can be explained by formation of Zn aggregates with carbonate or phosphate anions present in simulated gastrointestinal fluids (Table S2). It was demonstrated that dissolved Zn ions rapidly react with carbonate or phosphate ions at equivalent levels to form Zn_4_CO_3_(OH)_6_·H_2_O or Zn_3_(PO_4_)_2_·xH_2_O (x = 2 or 4), which are not soluble under alkaline condition [33]. Hence, dissolved Zn ions in the gastric fluid could form Zn-carbonate or Zn-phosphate aggregates in three consecutive digestion fluids, thereby reducing total dissolved Zn ions.

### 3.3. Interactions between ZnO NPs and Food Proteins

**Figure 3:**
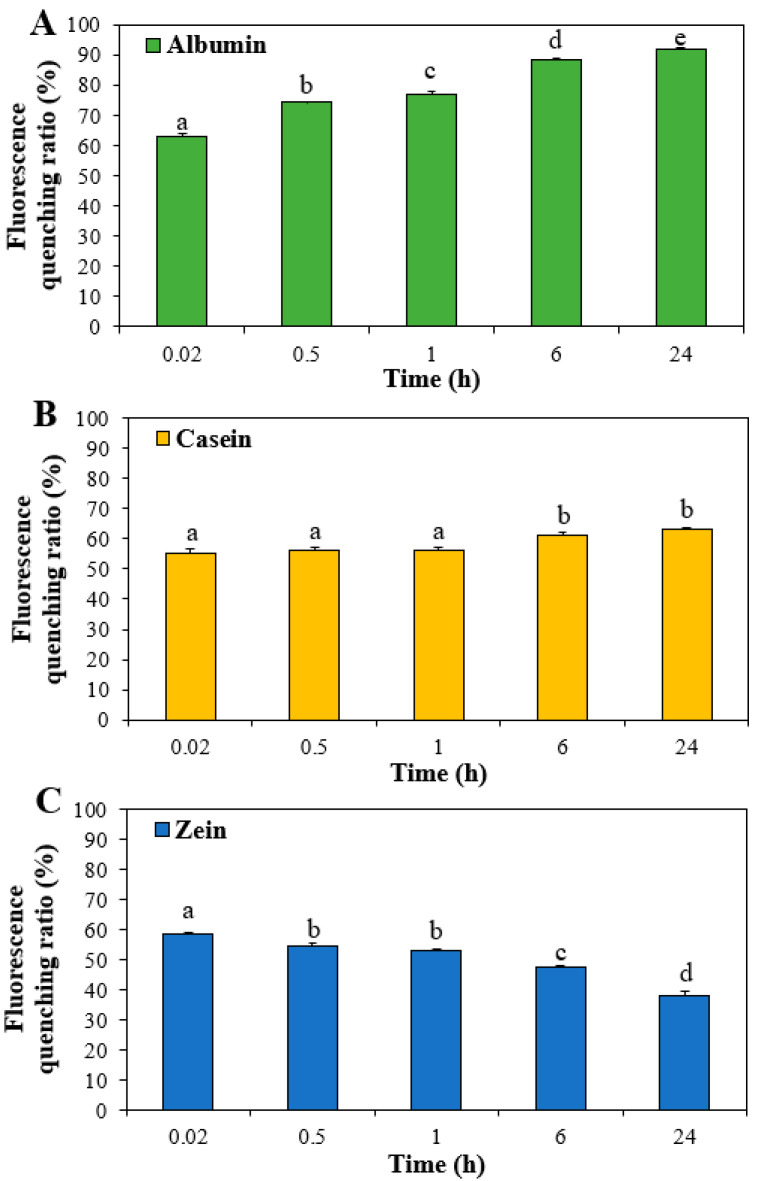
Fluorescence quenching ratios of (**A**) albumin, (**B**) casein, and (**C**) zein by interactions with ZnO NPs. Different lowercase letters (a–d) indicate significant differences among incubation times (*p* < 0.05).

Changes in the fluorescence of food proteins by interaction with ZnO NPs were analyzed since tryptophan residues in proteins exhibit maximum fluorescence at 340 nm. Figure 3 shows that fluorescence quenching (55–63%) of all proteins occurred just after incubation with ZnO NPs for 0.02 h, suggesting rapid interactions between ZnO NPs and food proteins. The highest fluorescence quenching of albumin among three food proteins was found by interaction with ZnO NPs, indicating high interaction between ZnO NPs and albumin. The fluorescence quenching ratios of albumin and casein significantly increased as incubation time increased. On the other hand, the quenching ratios of zein decreased after 0.5–24 h, which seems to be resulted from low solubility and large hydrodynamic diameters of zein under aqueous condition (Figure 1A), not really attributed to the interaction [25,26].

### 3.4. Primary Structural Stability and Digestion of Food Proteins by Interactions with ZnO NPs

**Figure 4:**
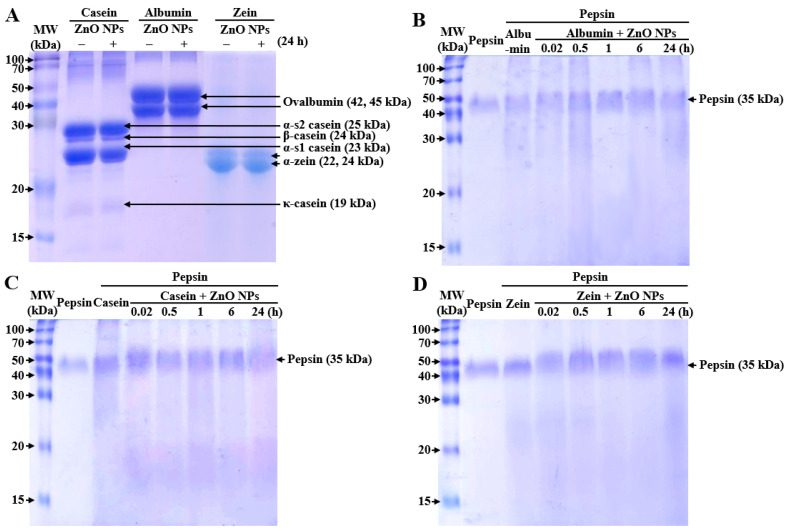
Sodium dodecyl sulfate–polyacrylamide gel electrophoresis (SDS–PAGE) of (**A**) food proteins interacted with ZnO NPs for 24 h, and (**B**) albumin, (**C**) casein, and (**D**) zein by interactions with ZnO NPs followed by pepsin treatment for 1 h. MW, molecular weight.

The interaction effect on the stability of primary structure of food proteins was evaluated by SDS–PAGE. Figure 4A shows that the primary structure of all proteins remained intact by interactions with ZnO NPs for 24 h. Moreover, albumin, casein, and zein were completely digested by pepsin treatment (Figure 4B–D). These results suggest that the interactions between ZnO NPs and food proteins did not affect primary structural stability nor digestion efficacy of the proteins.

### 3.5. Cytotoxicity of ZnO NPs by Interactions with Food Proteins

**Figure 5:**
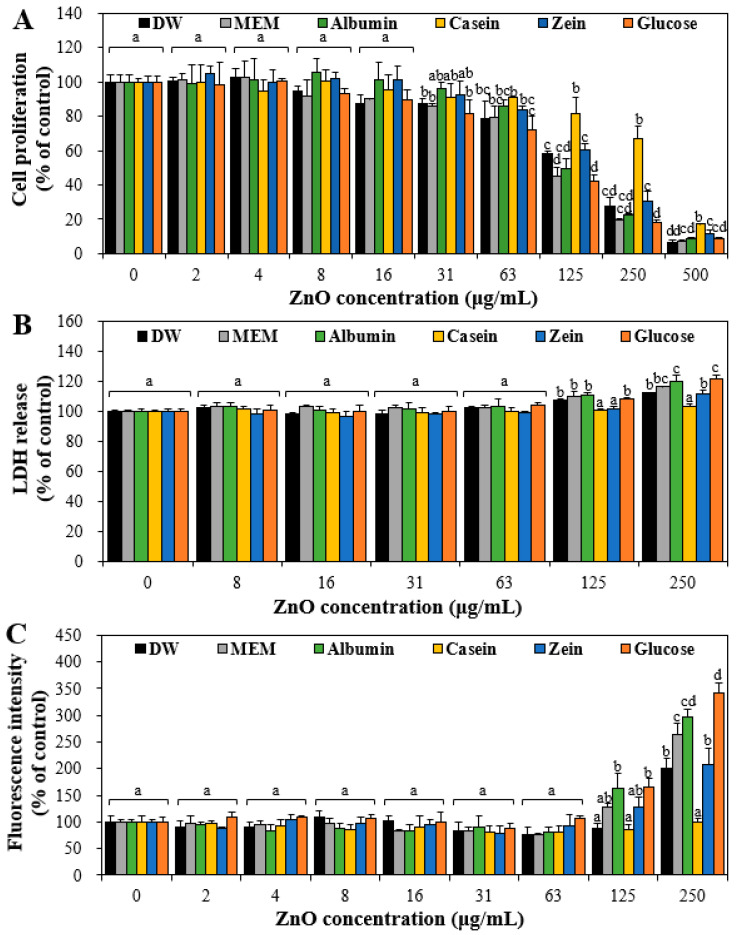
Effects of interactions between ZnO NPs and food proteins or glucose on (**A**) cell proliferation, (**B**) lactate dehydrogenase (LDH) release, and (**C**) reactive oxygen species (ROS) generation in Caco-2 cells after 24 h. Cells without ZnO NPs were used as controls. Different lowercase letters (a–d) indicate significant differences among control and ZnO NPs interacted with DW, MEM, food proteins, or glucose (*p* < 0.05).

The effect of the interactions on cytotoxicity was evaluated in terms of cell proliferation inhibition, LDH release, and ROS generation. The results demonstrate that ZnO NPs in food proteins or glucose significantly inhibited cell proliferation at more than 63 μg/mL (Figure 5A). However, ZnO NPs in casein or zein had statistically low effect on cell proliferation at 125–500 μg/mL. Significantly increased LDH release and ROS generation were induced by ZnO NPs in food matrices at more than 125μg/mL (Figure 5B,C). The highest LDH release and ROS generation were found when ZnO NPs were interacted with albumin or glucose, followed by interaction with MEM. Relatively low cytotoxicity of ZnO NPs in casein or zein was found in all cases, probably related to their large hydrodynamic diameters, namely, high aggregate fates (Figure 1A) and relatively weak protein–NP interactions (Figure 3). In other words, high interaction between ZnO NPs and albumin (Figure 3A) induced small hydrodynamic diameters (Figure 1A), which could increase the cytotoxicity of ZnO NPs (Figure 5). It is interesting to note that ZnO NPs in casein had no effects on LDH release (Figure 5B) and ROS generation (Figure 5C) even at the highest concentration tested and only inhibited cell proliferation (Figure 5A). It is probable that ZnO NPs in casein only cover the surface of cell membranes, thereby inhibiting cell proliferation without causing cell death or ROS generation. Hence, casein might be an effective matrix to reduce the cytotoxicity of ZnO NPs. All cytotoxicity results suggest the cytotoxicity of ZnO NPs could be modulated by interactions with food matrices.

### 3.6. Cellular Uptake of ZnO NPs by Interactions with Food Proteins

**Figure 6:**
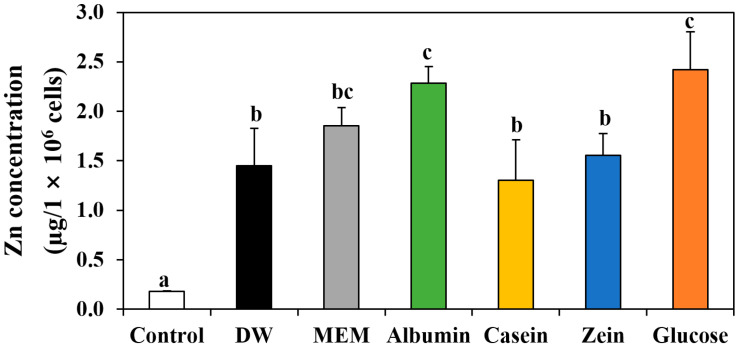
Effect of interactions between ZnO NPs and food proteins or glucose on cellular uptake in Caco-2 cells after 6 h, as determined by analyzing total intracellular Zn levels using inductively coupled plasma–atomic emission spectroscopy (ICP–AES). Cells without ZnO NPs were used as a control. Different lowercase letters (a–c) indicate significant differences among control and ZnO NPs interacted with DW, MEM, food proteins, or glucose (*p* < 0.05).

The effect of the interactions on cellular uptake of ZnO NPs was evaluated by measuring total intracellular Zn levels using ICP–AES. Figure 6 demonstrates that cellular uptake of ZnO NPs significantly enhanced by interactions with matrices, with an order of DW, casein, and zein < MEM < albumin and glucose. This result is very likely to be associated with small hydrodynamic diameters of ZnO NPs in albumin or glucose (Figure 1A), which facilitate efficient cellular internalization. Size-dependent cellular uptake of NPs has been well reported, showing efficient higher uptake of NPs than bulk-sized particles [34,35]. Based on DLS, cytotoxicity, fluorescence quenching, and cellular uptake results, albumin seems to strongly interact with ZnO NPs, leading to enhanced cellular uptake related to small hydrodynamic diameters. However, the high cellular uptake of ZnO NPs in albumin could cause high cytotoxicity (Figure 5). These results are highly consistent with those obtained by ZnO NPs in glucose in our previous report, demonstrating small hydrodynamic diameters, high cellular uptake, and high cytotoxicity of ZnO NPs in glucose compared with those in other saccharides [9].

### 3.7. Intestinal Transport of ZnO NPs by Interactions with Food Proteins

**Figure 7:**
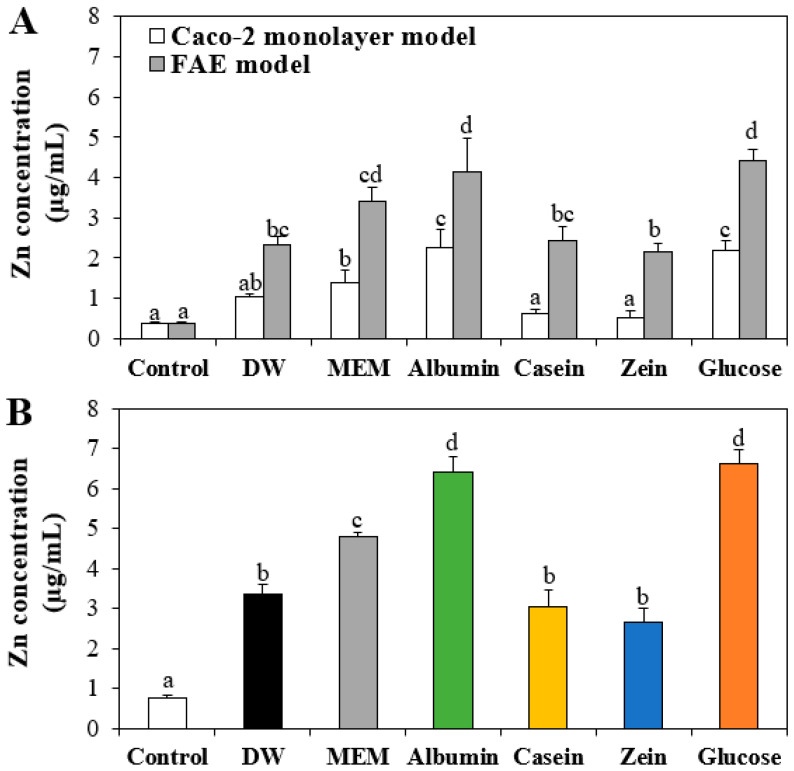
Effect of interactions between ZnO NPs and food proteins or glucose on (**A**) intestinal transport of ZnO NPs using in vitro models of Caco-2 monolayers and human follicle-associated epithelium (FAE) and (**B**) total combined intestinal transport amounts through both Caco-2 monolayer and FAE models after 6 h. Cells without ZnO NPs were used as controls. Different lowercase letters (a–d) indicate significant differences among control and ZnO NPs interacted with DW, MEM, food proteins, or glucose (*p* < 0.05).

The effect of the interactions on intestinal transport of ZnO NPs was evaluated using Caco-2 monolayer and FAE models, respectively. Intestinal transport amounts of ZnO NPs in food matrices by M cells were significantly higher than those by Caco-2 monolayers in all cases, indicating the major transport pathway of ZnO NPs by M cells (Figure 7A), which is in good agreement with our previous report [29,36]. On the other hand, ZnO NPs in albumin or glucose showed significantly high intestinal transportation compared with those in other matrices. When total transported levels though both Caco-2 monolayer and M cells were combined, the highest transport of ZnO NPs by interaction with albumin or glucose was confirmed, followed by interactions with MEM > DW, casein, and zein (Figure 7B). This result is highly consistent with the cellular uptake result (Figure 6). Hence, high interaction between ZnO NPs and albumin contributes towards exhibiting small hydrodynamic diameters, high cellular uptake, high cytotoxicity, and high intestinal transport, which were also reported by the interaction between ZnO NPs and glucose [9].

### 3.8. Toxicokinetics of ZnO NPs by Interactions with Food Matrices

**Figure 8:**
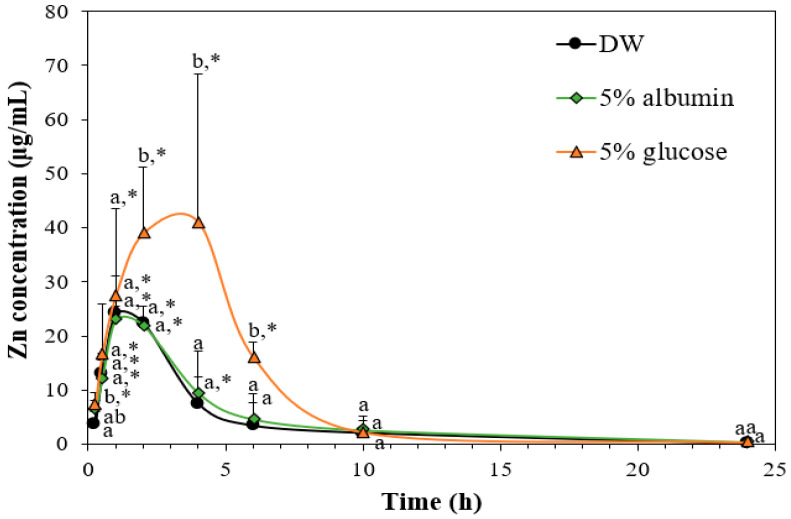
Effect of interactions between ZnO NPs and albumin or glucose on toxicokinetics of ZnO NPs after a single dose (100 mg/kg) oral administration in rats. Different lowercase letters (a,b) indicate significant differences among ZnO NPs interacted with DW, albumin, or glucose (*p* < 0.05). * Significant differences compared with untreated control (*p* < 0.05).

The effect of the interactions on toxicokinetics of ZnO NPs was evaluated after a single-dose oral administration in rats. Since only albumin among food proteins tested was found to significantly enhance cellular uptake and intestinal transport, the toxicokinetics of ZnO NPs in albumin was further evaluated and compared with those of ZnO NPs in glucose. The dose (100 mg/kg) was selected based on our previous report, showing no acute oral toxicity [37]. The result demonstrates that ZnO NPs in DW and albumin reached peak concentrations at 1.3 and 1.0 h, respectively, and returned to normal levels after 6 h (Figure 8). Meanwhile, the maximum concentration of ZnO NPs in glucose was found at 3.5 h and the concentration decreased to basal level after 10 h, showing higher absorption amount compared with those of ZnO in DW or albumin. Toxicokinetic parameters also confirm these results, showing significantly higher C_max_, T_max_, AUC, T_1/2_, MRT, CL/F, and absorption of ZnO NPs in glucose than those of ZnO in DW or albumin (Table 1). No significant differences in all toxicokinetic parameters between ZnO in DW and ZnO in albumin were found (*p* > 0.05). These results indicate that the interaction between ZnO NPs and glucose could enhance in vivo oral absorption as previously reported [8], but ZnO interaction with albumin did not affect toxicokinetic behaviors. These results are contradictory to in vitro cellular uptake and intestinal transport results (Figure 6 and Figure 7). It is probable that the interaction between ZnO NPs and albumin is not strong enough to enhance in vivo oral toxicokinetics, requiring further study on the interaction mechanisms.

### 3.9. Oral Toxicity of ZnO NPs by Interactions with Food Matrices

**Figure 9:**
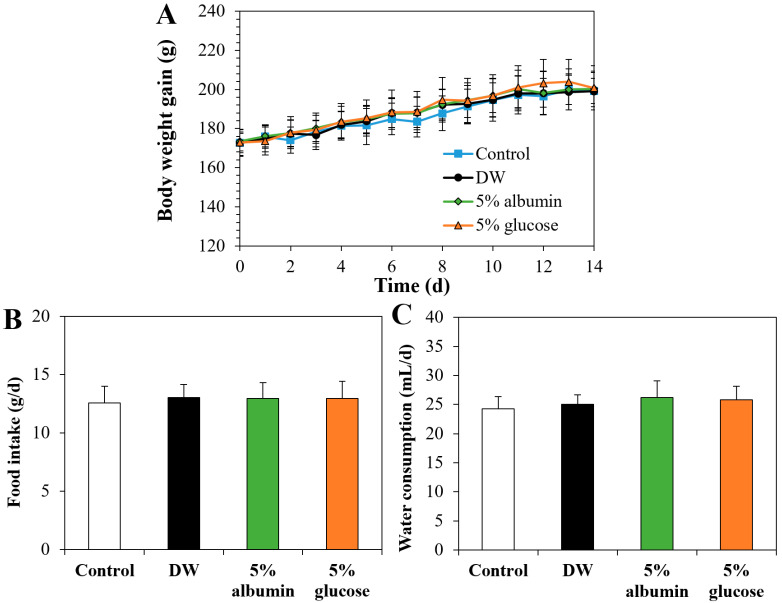
Changes in (**A**) body weight gains, (**B**) food intake, and (**C**) water consumption of rats after oral administration of ZnO NPs (100 mg/kg) interacted with DW, albumin, or glucose for 14 consecutive days. Rats administered an equivalent volume of DW without ZnO NPs were used as controls. No significant differences among untreated control and ZnO interacted with DW, albumin, or glucose were found (*p* > 0.05).

The effect of the interactions on oral toxicity of ZnO NPs was evaluated after oral administration in rats for 14 consecutive days. The most interacted protein with ZnO NPs, albumin, was selected for oral toxicity, together with glucose that was determined to enhance cellular uptake, intestinal transport, and oral absorption of ZnO NPs in this study and in our previous report [9]. The same dose (100 mg/kg) for toxicokinetics was used for oral toxicity study based on our previous research [36]. Figure 9 demonstrates that body weight gain, food intake, and water consumption were not significantly affected by ZnO NPs in DW, albumin, or glucose. No significant changes in organo-somatic indices among control and ZnO NPs in DW, albumin, or glucose were also found (Table 2), suggesting their low oral toxicity.

Hematological and coagulation time values reveal no significant increase or decrease in rats administered ZnO NPs in DW compared with those of non-treated control rats, except significant increase in eosinophils (EO) (Table 3). All hematological and coagulation time values were not significantly affected by interactions between ZnO NPs and albumin or glucose compared with those in the control group. Serum biochemical parameters show that total protein (TP), albumin (ALB), alkaline phosphatase (ALP), creatine (CREA), blood urea nitrogen (BUN), and calcium (CA) values decreased, whereas significantly increased inorganic phosphorus (IP) value was found in rats administered ZnO NPs in DW compared with those in non-treated controls (Table 4). Significant decrease in TP, ALB, ALP, and CA was also found in ZnO NPs in albumin or glucose-treated rats. Decreased triglyceride (TG) value was only observed in rats treated with ZnO NPs in albumin. It is worth noting that reduced TP, ALB, ALP, CREA, BUN, and TG values are not considered toxic, but they are rather related to individual variation [38,39]. Changes in CA and IP values by ZnO NPs in DW, albumin, or glucose seems to be minor.

**Figure 10:**
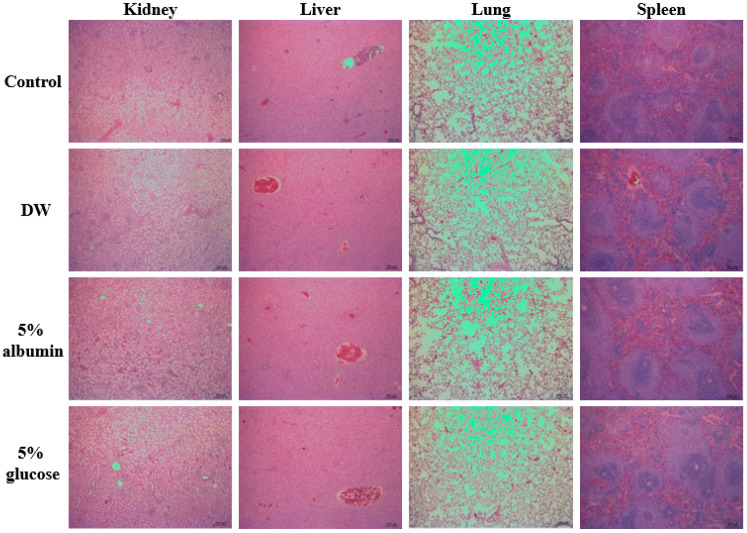
Normal histopathological sections of the kidney, liver, lung, and spleen of the rats after oral administration of ZnO NPs (100 mg/kg) interacted with DW, albumin, or glucose for 14 consecutive days. Images were magnified at 50×.

Histopathological findings show that minimal tubular degeneration and inflammatory cell infiltration or minimal cortical scar were observed, but only in one rat among five rats administered ZnO NPs in albumin or glucose (Table 5, Figure 10). Furthermore, the severity of the lesions was mild, and these histopathological findings are often observed as background lesions [40,41,42]. It is interesting to note that no abnormality was detected in rats treated with ZnO NPs in DW (Table 5, Figure 10). Taken together, ZnO NPs in DW did not cause acute oral toxicity at dose level tested, and the interactions between ZnO NPs albumin or glucose did not induce higher oral toxicity than ZnO NPs in DW. Similar toxicokinetic behaviors of ZnO NPs in DW to those of ZnO NPs in albumin could explain these oral toxicity results (Figure 8, Table 2). Moreover, it is likely that enhanced oral absorption amount of ZnO NPs in glucose was not enough to affect oral toxicity of ZnO NPs. It is also probable that the interaction was not strong enough to affect in vivo acute oral toxicity of ZnO NPs. Further study is required to determine the interaction mechanism between ZnO NPs and food matrices and to ascertain the interaction effects on chronic oral toxicity of ZnO NPs.

## 4. Conclusions

The interactions between ZnO NPs and food proteins such as albumin, casein, and zein were investigated and the interactions were compared with that with glucose. The interactions rapidly occurred as evidenced by high fluorescence quenching of all proteins and changes in zeta potentials of ZnO NPs in the presence of proteins, but differently depending on protein types. The interaction between ZnO NPs and albumin reduced hydrodynamic diameters, causing high cytotoxicity, cellular uptake, and intestinal transport in a similar manner to ZnO NPs interacted with glucose. The interactions between ZnO NPs and casein or zein induced high aggregates and reduced cytotoxicity without affecting in vitro cellular uptake and intestinal transport amounts. Oral absorption and toxicokinetic behaviors of ZnO NPs were not affected by interaction with albumin, contrary to their enhanced oral absorption by interaction with glucose. However, these interactions did not cause acute toxicity after oral administration in rats for 14 consecutive days, suggesting low interaction effect on in vivo acute oral toxicity. Further study is required to determine the interaction mechanism between ZnO NPs and food matrices and to ascertain the effect of the interactions between ZnO NPs and food matrices on chronic oral toxicity.

## Figures and Tables

**Table 1:** Toxicokinetic parameters and oral absorption after a single dose (100 mg/kg) oral administration of ZnO NPs interacted with DW, albumin, or glucose in rats.

Toxicokinetic Parameters	DW	5% Albumin	5% Glucose
C_max_ (µg/mL)	25.3 ± 3.1 ^a^	25.3 ± 2.1 ^a^	41.9 ± 4.6 ^b^
T_max_ (h)	1.3 ± 0.5 ^a^	1.0 ± 0.0 ^a^	3.5 ± 1.0 ^b^
AUC (h × µg/mL)	132.8 ± 27.1 ^a^	126.5 ± 18.2 ^a^	280.9 ± 49.8 ^b^
T_1/2_ (h)	2.5 ± 0.0 ^a^	2.7 ± 0.2 ^a^	4.5 ± 0.4 ^b^
MRT (h)	4.0 ± 0.4 ^a^	4.3 ± 0.2 ^a^	5.8 ± 0.7 ^b^
CL/F (mL/h)	625.2 ± 151.1 ^a^	641.1 ± 82.3 ^a^	256.6 ± 24.0 ^b^
Absorption (%)	8.9 ± 1.9 ^a^	8.4 ± 0.3 ^a^	18.8 ± 3.1 ^b^

Different lower–case letters (^a,b^) indicate significant differences among ZnO NPs interacted with DW, albumin, or glucose (*p* < 0.05). DW, distilled water; C_max_, maximum concentration; T_max_, time to maximum concentration; AUC, area under the plasma concentration-time curve; T_1/2_, half-life; MRT, mean residence time; CL/F, apparent clearance.

**Table 2:** Organo-somatic indices of rats after oral administration of ZnO NPs (100 mg/kg) interacted with DW, albumin, or glucose for 14 consecutive days.

Organ	Control	DW	5% Albumin	5% Glucose
Brain	0.8 ± 0.0	0.8 ± 0.1	0.8 ± 0.1	0.8 ± 0.1
Heart	0.4 ± 0.0	0.4 ± 0.0	0.4 ± 0.0	0.4 ± 0.0
Kidney	0.8 ± 0.1	0.8 ± 0.0	0.8 ± 0.1	0.8 ± 0.0
Large intestine	1.9 ± 0.3	1.6 ± 0.3	1.7 ± 0.3	1.5 ± 0.6
Liver	3.7 ± 0.2	3.8 ± 0.3	3.9 ± 0.3	3.9 ± 0.2
Lung	0.6 ± 0.1	0.6 ± 0.1	0.6 ± 0.1	0.6 ± 0.1
Ovary	0.1 ± 0.0	0.1 ± 0.0	0.1 ± 0.0	0.1 ± 0.0
Small intestine	4.1 ± 0.4	4.2 ± 0.3	4.3 ± 0.3	4.1 ± 0.3
Spleen	0.3 ± 0.1	0.3 ± 0.0	0.3 ± 0.0	0.3 ± 0.1
Stomach	1.7 ± 0.3	1.7 ± 0.6	1.5 ± 0.4	1.3 ± 0.3

No significant differences among untreated control, ZnO interacted with DW, albumin, or glucose were found (*p* > 0.05).

**Table 3:** Hematological and coagulation time values of rats after oral administration of ZnO NPs (100 mg/kg) interacted with DW, albumin, or glucose for 14 consecutive days.

Groups	WBC	WBC Differential Counting (%)					RBC	Hb	HCT	MCV	MCH	MCHC	RETI	PLT	PT	APTT
	(10^3^/μL)	NE	LY	MO	EO	BA	(10^6^/μL)	(g/dL)	(%)	(fL)	(pg)	(g/dL)	(%)	(10^3^/μL)	(s)	(s)
**Control**	6.89 ± 0.86	10.3 ± 1.9	85.0 ± 1.9	1.9 ± 0.3	1.3 ± 0.4	0.6 ± 0.1	7.08 ± 0.19	13.8 ± 0.5	44.4 ± 1.7	62.6 ± 1.4	19.5 ± 0.4	31.1 ± 0.5	3.25 ± 0.88	950 ± 151	17.4 ± 0.5	38.5 ± 2.7
**DW**	6.47 ± 1.21	14.6 ± 5.0	78.3 ± 5.4	2.0 ± 0.3	3.1 ± 0.9 **	0.8 ± 0.3	7.04 ± 0.41	13.6 ± 1.0	43.8 ± 2.9	62.2 ± 1.6	19.3 ± 0.5	31.0 ± 0.6	2.72 ± 0.22	902 ± 300	17.3 ± 0.9	39.0 ± 7.3
**5% albumin**	6.74 ± 1.22	7.9 ± 1.8	86.4 ± 1.5	2.0 ± 0.2	1.8 ± 0.4	0.7 ± 0.4	7.27 ± 0.34	14.0 ± 0.5	45.4 ± 1.3	62.5 ± 1.3	19.2 ± 0.4	30.8 ± 0.4	2.65 ± 0.55	1056 ± 76	17.1 ± 0.3	46.2 ± 4.8
**5% glucose**	6.79 ± 1.37	9.5 ± 1.2	83.7 ± 1.1	2.1 ± 0.5	2.2 ± 0.4	0.8 ± 0.4	7.25 ± 0.33	13.9 ± 0.6	44.5 ± 2.0	61.5 ± 1.1	19.2 ± 0.5	31.2 ± 0.6	2.97 ± 0.60	1171 ± 121	16.7 ± 0.3	40.4 ± 13.1

** Significant differences compared with non-treated control group at *p* < 0.01. WBC, white blood cell; NE, neutrophils; LY, lymphocytes; MO, monocytes; EO, eosinophils; BA, basophils; RBC, red blood cell; Hb, hemoglobin, HCT, hematocrit; MCV, mean corpuscular volume; MCH, mean corpuscular hemoglobin; MCHC, mean corpuscular hemoglobin concentration; RETI, reticulocyte; PLT, platelet; PT, prothrombin time; APTT, activated partial thromboplastin time.

**Table 4:** Serum biochemical values of rats after oral administration of ZnO NPs (100 mg/kg) interacted with DW, albumin, or glucose for 14 consecutive days.

Groups	TP	ALB	A/G	T-BIL	ALP	AST	ALT	CREA	BUN	CHOL	TG	GLU	CA	IP	CK	Na	K	Cl
	(g/dL)	(g/dL)		(mg/dL)	(U/L)	(U/L)	(U/L)	(g/dL)	(g/dL)	(g/dL)	(g/dL)	(g/dL)	(g/dL)	(g/dL)	(IU/L)	(mmol/L)	(mmol/L)	(mmol/L)
**Control**	6.8 ± 0.2	4.4 ± 0.1	1.9 ± 0.1	0.0 ± 0.0	783 ± 131	77 ± 6	49 ± 5	0.54 ± 0.04	26.3 ± 4.7	88 ± 14	82 ± 9	299 ± 33	12.7 ± 0.3	9.4 ± 0.7	185 ± 86	147.7 ± 0.7	5.60 ± 0.30	97.4 ± 0.9
**DW**	6.2 ± 0.1 ^**^	4.1 ± 0.1 ^**^	2.0 ± 0.1	0.0 ± 0.0	550 ± 63 ^*^	72 ± 5	48 ± 4	0.48 ± 0.02 ^*^	19.6 ± 2.8 ^*^	90 ± 8	66 ± 15	289 ± 18	12.1 ± 0.3 ^*^	10.9 ± 0.8 ^*^	278 ± 229	147.2 ± 1.0	6.22 ± 0.22	97.9 ± 1.2
**5% albumin**	6.2 ± 0.2 ^**^	4.1 ± 0.2 ^**^	1.9 ± 0.1	0.0 ± 0.0	542 ± 128 ^*^	74 ± 7	56 ± 9	0.49 ± 0.03	25.4 ± 2.5	83 ± 8	51 ± 13 ^*^	247 ± 39	12.1 ± 0.3 ^*^	10.3 ± 0.8	191 ± 45	148.0 ± 1.6	5.84 ± 0.46	98.8 ± 1.2
**5% glucose**	6.1 ± 0.2 ^**^	3.9 ± 0.2 ^**^	1.8 ± 0.2	0.0 ± 0.0	592 ± 114	71 ± 10	48 ± 6	0.50 ± 0.02	20.0 ± 2.9	85 ± 8	62 ± 16	259 ± 17	12.0 ± 0.2 ^*^	10.4 ± 0.8	209 ± 39	147.3 ± 1.7	5.88 ± 0.40	98.7 ± 2.1

* and ** indicate significant differences compared with non-treated control group at *p* < 0.05 and *p* < 0.01, respectively. TP, total protein; ALB, albumin; A/G, albumin/globulin ratio; T-BIL, total bilirubin; ALP, alkaline phosphatase; AST, aspartate aminotransferase; ALT, alanine aminotransferase; CREA, creatine; BUN, blood urea nitrogen; CHOL, total cholesterol; TG, triglycerides; GLU, glucose; CA, calcium; IP, inorganic phosphorus; CK, creatine kinase; Na, sodium; K, potassium; Cl, chloride.

**Table 5:** Summary of histopathological findings of rats after oral administration of ZnO NPs (100 mg/kg) interacted with DW, albumin, or glucose for 14 consecutive days.

Organs	Numbers of Animals	Histopathological Findings	Control	DW	5% Albumin	5% Glucose
Liver	5	No abnormalities detected	5	5	5	5
Kidney	5	No abnormalities detected	5	5	4	4
		Tubular degeneration and inflammatory cell infiltration minimal	0	0	0	1
		Scar, cortical minimal	0	0	1	0
Lung	5	No abnormalities detected	5	5	5	5
Spleen	5	No abnormalities detected	5	5	5	5

## Data Availability

The data presented in this study are available in the article and Supplementary Materials.

## Supplementary Materials

The following are available online at https://www.mdpi.com/article/10.3390/nano11112922/s1, Figure S1: Scanning electron microscopy (SEM) images and size distribution of ZnO NPs, Table S1: Composition of artificial lysosomal fluid (ALF), Table S2, Composition of simulated digestion fluids.
