# Supplementary material for: Interaction between ZnO Nanoparticles and Albumin and Its Effect on Cytotoxicity, Cellular Uptake, Intestinal Transport, Toxicokinetics, and Acute Oral Toxicity
Source: Nanomaterials (Basel). 2021 Oct 31;11(11):2922. doi: 10.3390/nano11112922 (PMC8625151; doi:10.3390/nano11112922)
Supplement: Supplementary file 1 [file nanomaterials-11-02922-s001.zip › nanomaterials-1421050-SI modify.pdf]

nanomaterials

MDPI

Supplementary Materials

Interaction between ZnO nanoparticles and albumin causes high cytotoxicity, cellular uptake, and intestinal transport, but does not cause acute oral toxicity

Eun-Been Jung, Jin Yu, and Soo-Jin Choi *

Division of Applied Food System, Major of Food Science & Technology, Seoul Women's University, Seoul 01797, Republic of Korea; ky5031@swu.ac.kr (J.Y.); ebe2@swu.ac.kr (E.-B.J.); ky5031@swu.ac.kr (J.Y.)
* Correspondence: sjchoi@swu.ac.kr; Tel.: +82-2-970-5634

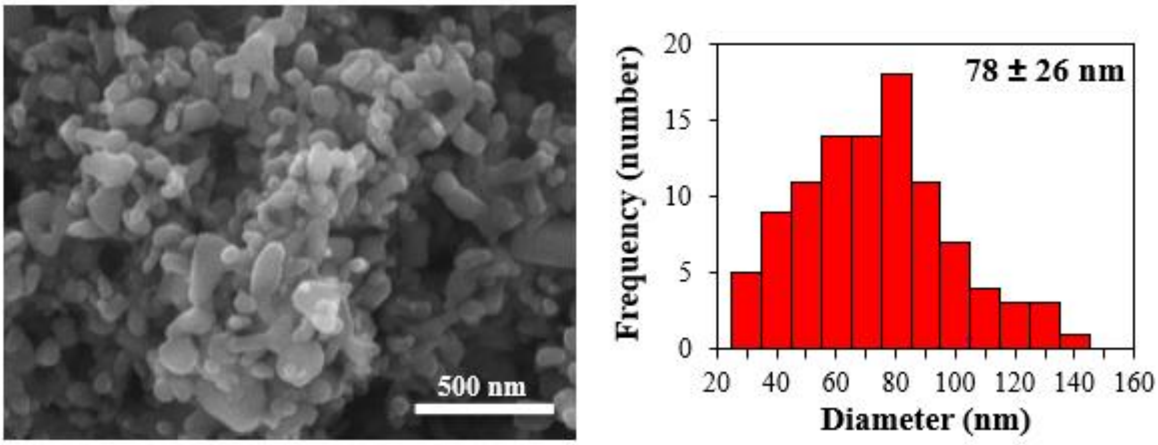

Figure S1. Scanning electron microscopy (SEM) images and size distribution of ZnO NPs. Particle size distribution was determined by randomly selecting more than 100 particles from the SEM images.

Table S1. Composition of artificial lysosomal fluid (ALF).

pH	Composition	Amounts/L
4.5	Sodium chloride	3.2 g/L
	Sodium hydroxide	6.0 g/L
	Citric acid	20.8 g/L
	Calcium chloride dihydrate	128 mg/L
	Sodium phosphate	71 mg/L
	Sodium sulfate	39 mg/L
	Magnesium chloride hexahydrate	106 mg/L
	Glycerin	59 mg/L
	Sodium citrate dihydrate	77 mg/L
	Sodium tartrate dihydrate	90 mg/L
	Sodium lactate	52 mg/L
	Sodium pyruvate	86 mg/L
	Formaldehyde	1 mL
	Glycine	30.3 g/L

Table S2. Composition of simulated digestion fluids.

Digestion fluids	pH	Composition	Amounts/L
Saliva	6.8 ± 0.1	α-amylase	290 mg/L
		Potassium chloride	896 mg/L
		Potassium thiocyanate	200 mg/L
		Mucin	25 mg/L
		Sodium sulfate	570 mg/L
		Sodium chloride	298 mg/L
		Sodium phosphate monobasic dihydrate	1.1 g/L
		Sodium bicarbonate	1.7 g/L
		Urea	200 mg/L
		Uric acid	15 mg/L
Gastric fluid	1.3 ± 0.1	Bovine serum albumin	1 g/L
		Calcium chloride dihydrate	400 mg/L
		Glucosaminhydrochloride	330 mg/L
		Glucose	650 mg/L
		Glucuronic acid	20 mg/L
		Potassium chloride	824 mg/L
		Mucin	3 g/L
		Sodium chloride	2.8 g/L
		Sodium dihydrogen phosphate dihydrate	333 mg/L
		Pepsin	2.5 g/L
		Urea	85 mg/L
Duodenal fluid	8.1 ± 0.1	Bovine serum albumin	1 g/L
		Calcium chloride dihydrate	200 mg/L
		Hydrogen chloride	79 mg/L
		Potassium chloride	564 mg/L
		Potassium dihydrogen phosphate	80 mg/L
		Lipase	1.5 g/L
		Magnesium chloride	22.7 mg/L
		Sodium chloride	7 g/L
		Sodium bicarbonate	3.4 g/L
		Pancreatin	9 g/L
		Urea	100 mg/L
Bile fluid	8.2 ± 0.1	Bile extract	30 g/L
		Bovine serum albumin	1.8 g/L
		Calcium chloride dihydrate	222 mg/L
		Hydrogen chloride	66 mg/L
		Potassium chloride	376 mg/L
		Sodium chloride	5.3 g/L
		Sodium bicarbonate	5.8 g/L
		Urea	250 mg/L
